# Interleukin-17A Interweaves the Skeletal and Immune Systems

**DOI:** 10.3389/fimmu.2020.625034

**Published:** 2021-02-04

**Authors:** Mengjia Tang, Lingyun Lu, Xijie Yu

**Affiliations:** ^1^ Department of Endocrinology and Metabolism, Laboratory of Endocrinology and Metabolism, National Clinical Research Center for Geriatrics, West China Hospital, Sichuan University, Chengdu, China; ^2^ Department of Integrated Traditional Chinese and Western Medicine, Laboratory of Endocrinology and Metabolism, West China Hospital, Sichuan University, Chengdu, China

**Keywords:** osteoimmunology, interleukin-17A, osteoclasts, osteoblasts, postmenopausal osteoporosis, rheumatoid arthritis, psoriatic arthritis, axial spondyloarthritis

## Abstract

The complex crosstalk between the immune and the skeletal systems plays an indispensable role in the maintenance of skeletal homeostasis. Various cytokines are involved, including interleukin (IL)-17A. A variety of immune and inflammatory cells produces IL-17A, especially Th17 cells, a subtype of CD4^+^ T cells. IL-17A orchestrates diverse inflammatory and immune processes. IL-17A induces direct and indirect effects on osteoclasts. The dual role of IL-17A on osteoclasts partly depends on its concentrations and interactions with other factors. Interestingly, IL-17A exerts a dual role in osteoblasts *in vitro*. IL-17A is a bone-destroying cytokine in numerous immune-mediated bone diseases including postmenopausal osteoporosis (PMOP), rheumatoid arthritis (RA), psoriatic arthritis (PsA) and axial spondylarthritis (axSpA). This review will summarize and discuss the pathophysiological roles of IL-17A on the skeletal system and its potential strategies for application in immune-mediated bone diseases.

## Introduction

Over the last 20 years, a growing body of research has focused on the relationship between the skeletal and immune systems. Subsequently, the term “osteoimmunology” was defined for this field of study. Accumulating evidence has shown that multiple components of immune systems including immune organs, multiple immune cells, and immune factors, participate in bone metabolism. In turn, bone cells, including osteoclasts, osteoblasts, bone lining cells, and osteocytes, are indispensable for the regulation of immune systems. The interaction between the skeletal and immune systems constitutes a complex network and is involved in the pathological process of many immune-mediated bone diseases. Recent studies have shown that IL-17A as one of the immune-derived cytokines participates in the regulation of bone metabolism. Understanding the effect of IL-17A on bone metabolism is more conducive to develop new-targeted drugs for immune-related bone diseases. This review will summarize the current knowledge of IL-17A in the skeletal system and will discuss the potential clinical value of IL-17A in immune-mediated bone diseases.

## IL-17A Signaling Pathway and Function

The IL-17 family includes six major isoforms: IL-17A, IL-17B, IL-17C, IL-17D, IL-17E, and IL-17F. Six of these isoforms interact with the five receptors (IL-17RA-E), respectively (1). IL-17A was the first member discovered and the most studied of the IL-17 family. Thereafter, following large-scale sequencing of the human and other vertebrate genomes, additional isoforms homologous to IL-17A were found (2). In 1993, Rouvier et al. cloned IL-17 for the first time. IL-17 was initially called the murine cytotoxic T lymphocyte-associated antigen-8 (mCTLA8) and was found to share 57% homology with the open reading frame 13 (ORF13) of Herpesvirus saimiri (HVS) (3). Subsequently, Yao et al. and Fossiez et al. cloned IL-17A in 1995 and 1996, respectively. Humans and mice share 25% amino acid sequence homology in IL-17A (4). IL-17A has been reported to be involved in inflammation and hematopoiesis and its secretion might be restricted to activated memory CD4 ^+^ T cells (4, 5). Current studies indicate that IL-17A is mainly produced by a special CD4^+^ T cell subtype, Th17 cells (6). In addition, other types of lymphocytes including IL-17^+^ CD8^+^ T cells (Tc17 cells) (7), invariant natural killer T (8), Foxp3^+^ Treg cells (9), γδ T cells (10), lymphoid−tissue inducer (LTi)−like cells (11), innate lymphoid cell (ILC3) (12), and NK cells can produce IL-17A. Besides, lymphocytes, myeloid cells including macrophages/monocytes (13), neutrophils (14), mast cells (15), Paneth cells (16) can secret IL-17A. Moreover, fibroblasts can also produce IL-17A (17). Multiple cytokines affect the expression of IL-17A, IL-1β, tumor necrosis factor (TNF)-β, IL-21, and IL-23 stimulate the expression of IL-17A in T cells (18), while interferon (IFN)-α inhibits the expression of IL-17A in T cells (19). Thus, IL-17A is derived from a variety of immune and inflammatory cells and its expression is regulated by a variety of immune factors.

IL-17A interacts with its receptors to activate downstream regulators and trigger cellular responses. Receptors for IL-17A are ubiquitously expressed on the cellular surface including synoviocytes, chondrocytes, fibroblasts, monocytes/macrophages, mast cells (20, 21). Bone cells including osteoclasts and osteoblasts also express IL-17RA (22). The interaction between IL-17RA and IL-17RC forms a complex to mediate the functions of IL-17A. The binding of IL-17A to the related receptor sites of IL-17RA alters the affinity and specificity of the symmetry receptor site. This response promotes the form of IL-17RA/RC heterodimer and makes an optimal response to mediate the functions of IL-17A homodimers (23, 24). Both IL-17RA and IL-17RC are type I transmembrane proteins. IL-17RA includes two extracellular fibronectin II-like domains and two intracellular “SEFIR” domains (25, 26). The SEFIR is homologous to Toll-IL-1R (TIR) domains found in the TLR/IL-1R family and is crucial for triggering downstream signaling events. IL-17A binds to its heterodimeric receptors complex and then recruits Act1 to activate classic IL-17A signaling cascades through receptor-associated factor 6 (TRAF6) proteins. TRAF6 binding subsequently triggers the mitogen-activated protein kinase (MAPK) pathway, extracellular signal–regulated kinase 1/2 (ERK1/2) pathway, and nuclear factor-κB (NF-κB) pathway. Among the non-classical signaling pathways, IL-17A integrates with epidermal growth factor receptor (EGFR), Notch 1, homolog translocation-associated (NOTCH1), C-type lectin receptor components, and interacts with fibroblast growth factor (FGF) signaling to initiate downstream biological responses (27).

In physiological conditions, IL-17A, as an immune and inflammatory-related factor, plays a protective role in host defenses against many bacterial and fungal pathogens (28). IL-17A activates neutrophils to promote neutrophil recruitment and accumulation (29). Meanwhile, IL-17A also affects the activity of B and T cells to act as a bridge between innate and acquired immune responses. Many studies have suggested that IL-17A is involved in the pathophysiological process of multiple diseases, including inflammatory bowel disease, breast cancer (30), lung cancer (31), cardiovascular system (32), uveitis (33), rheumatoid arthritis (RA), and psoriasis.

## Effects of IL-17A on the Skeletal System

### Osteoclasts

**Figure 1:**
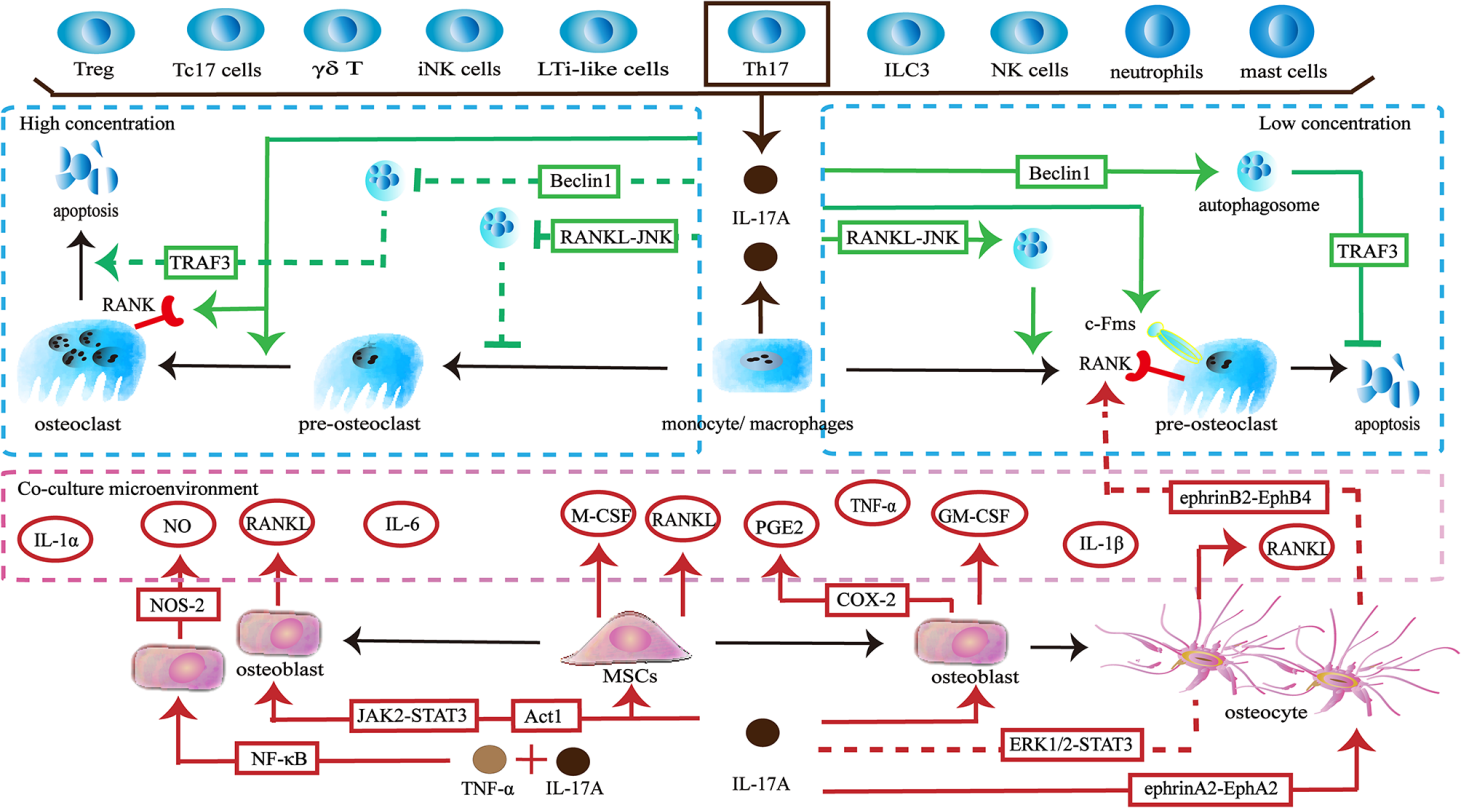
Effects of IL-17A on osteoclasts. ① IL-17A is produced by multiple lymphocytes including Treg cells, IL-17^+^ CD8^+^ T cells (Tc17 cells), γδ T cells, invariant natural killer T (iNK cells), and Th17 cells. Myeloid cells including macrophages/monocytes, neutrophils and mast cells can also secrete IL-17A. Th17 cells are the main source of IL-17A. ② Low concentrations of IL-17A promote osteoclastogenesis through the RANKL-JNK signaling pathway and reduces the apoptosis of osteoclasts through the RANKL-Beclin1-autophagy-TRAF3 pathway. IL-17A increases the expression of c-Fms in osteoclast precursors to promote proliferation and differentiation. IL-17A increases the number of RANK^+^ osteoclast precursors to influence subsequent RANKL-dependent osteoclastogenesis. ③ High concentration of IL-17A (5–50 ng/ml) inhibits osteoclastogenesis through the RANKL-JNK signaling pathway and promotes the apoptosis of osteoclasts through the RANKL-Beclin1-autophagy-TRAF3 pathway. However, a higher concentration of IL-17A (100 ng/ml) increases the number of RANK+ osteoclast precursors and induces polynuclear osteoclast formation. ④ IL-17A acts on osteoclast-supporting cells including mesenchymal stem cells, osteoblasts and osteocytes to produce various cytokines and molecules to regulate osteoclastogenesis indirectly.

The skeleton maintains physiological function through a dynamic balance of bone formation and resorption. Osteoclasts derive from the monocyte/macrophage lineage and are key players in bone resorption. IL-17A acts directly on osteoclast precursors. Exposure to IL-17A (0.1–1 ng/ml) induces the expression of colony-stimulating factor-1 receptor (c-Fms) and receptor activator of nuclear factor-κB (RANK) on human peripheral blood mononuclear cells (hPBMCs), thereby promoting more hPBMCs to differentiate into functional osteoclasts. The effect is not dose-dependent, but 1 ng/ml of IL-17A shows the best induction (34). The direct effect of IL-17A on osteoclast precursors seems to be dependent on its concentration. A low concentration of IL-17A (0.5ng/ml) promotes autophagy of osteoclast precursors by activating the RANKL-JNK signaling pathway, thereby enhancing RANKL-induced osteoclast differentiation. However, treatment with a high concentration of IL-17A (5–50 ng/ml) inhibits autophagy and decreases osteoclast formation (35). In addition, a low level of IL-17A can reduce the apoptosis of osteoclasts and thus increases the number of osteoclasts by targeting the RANKL-Beclin1-autophagy-TRAF3 pathway (36). In turn, high levels of IL-17A increase apoptosis of osteoclasts and ultimately reduce pro-osteoclast mediators including cathepsin K, tartrate-resistant acid phosphatase (TRAP), and matrix metalloproteinase (MMP)-9 (36, 37). Interestingly, a higher concentration of IL-17A (100 ng/ml) promotes RANKL-induced polynuclear osteoclast formation and increases the expression of RANK and TRAP (38) (**Figure 1**).

Conversely, IL-17A can regulate osteoclast formation by targeting osteoclast-supporting cells. When activated by IL-17A, human bone marrow-derived mesenchymal stem cells (hBM-MSCs) secrete M-CSF and RANKL, thereby supporting osteoclastogenesis (39). When IL-17A binds to its receptors IL-17RA SEFIR/TILL domain on pre-osteoclasts, they trigger Act1 adaptor protein and may activate downstream JAK2-STAT3 signaling to promote the expression of RANKL (40–43). The upregulation of RANKL and the increase the ratio of RANKL/osteoprotegerin (OPG) promotes osteoclastogenesis (44, 45). Moreover, IL-17A stimulates osteoblast precursors to produce cyclooxygenase-2 (COX-2) related-prostaglandin E2 (PGE2), which is a facilitated factor in osteoclasts formation (46). The synergistic effects of IL-17A and TNF-α activate NF-κB-dependent pathways to promote the production of nitric oxide synthase-2 (NOS-2) and nitric oxide (NO). NO triggers the RANKL-RANK pathway to increase osteoclastic bone resorption (47). In addition, IL-17A and TNF-α synergistically induce osteoblast precursors to produce inflammatory factors including IL-1α, IL-1β and IL-6. These cytokines can up-regulate osteoclast activity (48). When activated by IL-17A, osteocytes inhibit the ERK1/2-STAT3 pathway and increase the RANKL/OPG ratio and TNF-α, thereby enhancing osteoclast formation. Furthermore, due to the activation of reversed ephrinA2-EphA2 signaling and suppression of ephrinB2-EphB4 signaling between osteocytes and osteoclast precursors, RANK^+^ bone marrow macrophages (BMMs) are increased, which influences subsequent RANKL-dependent osteoclastogenesis (49). In addition to providing osteoclastic activating factors, IL-17A can promote the expression of inhibitory factors. IL-17A promotes osteoblasts to produce granulocyte-macrophage colony-stimulating factor (GM-CSF), which in turn reduces the expression of RANK in osteoclast precursors and thus may weaken RANKL-RANK signaling to inhibit osteoclastogenesis (50). Moreover, GM-CSF maintains monocytes in an undifferentiated state by downregulating c-Fos, Fra-1, and nuclear factor of activated T cells 1 (Nfatc1) (51) (**Figure 1**).

The *in vitro* effects of IL-17A on osteoclasts are dual. Recent findings indicate that the direct effects of IL-17A on osteoclastogenesis are related to its concentration, but are not dose-dependent. Low concentration of IL-17A promotes osteoclastogenesis, while IL-17A begins to inhibit the formation of osteoclasts as the concentration increases. Strangely, further increases in the concentrations of IL-17A promote osteoclastogenesis. The precise relationship requires further exploration. In addition, IL-17A is involved in osteoclastogenesis *via* other types of cells and factors. The integrated network of cells and the factors they produce makes the specific effects attributable to IL-17A difficult to determine. The dominant effect may vary in different states. Thus, the role of IL-17A needs to be explored in more complex environments *in vivo*.

### Osteoblasts

The osteoblast is another important player involved in maintaining bone homeostasis. When IL-17A binds receptors on pre-osteoblasts, it promotes their proliferation in a dose-dependent manner (49, 52, 53). When IL-17A activates TRAF6 and Act1 to initiate Ras-related C3 botulinum toxin substrate 1 guanosine triphosphatase (Rac1 GTPase) and NADPH oxidase 1 (Nox1), the expression of reactive oxygen species (ROS) is upregulated to promote pre-osteoblasts proliferation (39).

**Figure 2:**
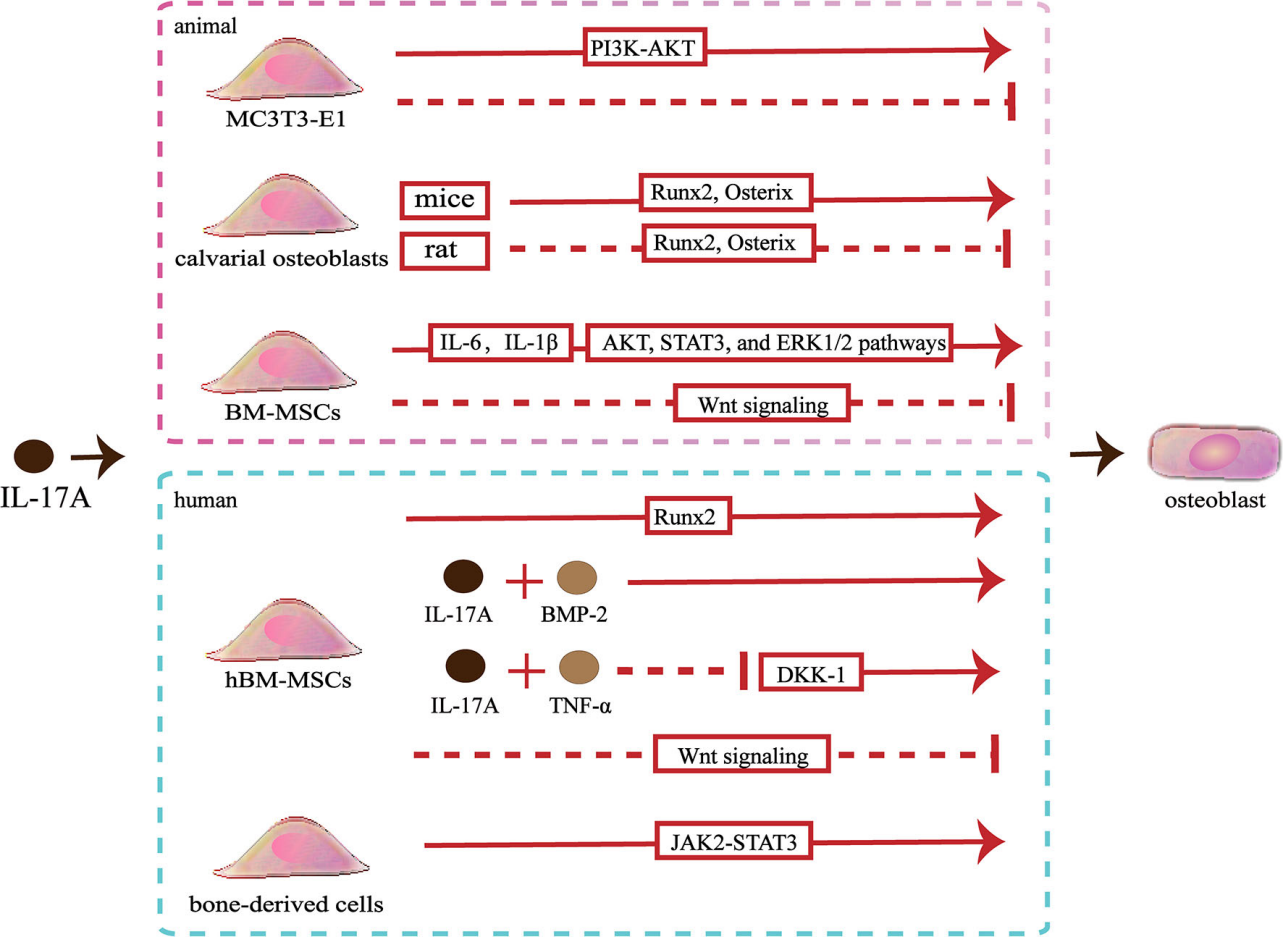
Effects of IL-17A on osteoblasts. ①IL−17A promotes MC3T3-E1 to differentiate into osteoblasts by activating the PI3K-AKT signaling pathways, whereas another study indicated that IL-17A inhibited osteoblastic differentiation of MC3T3-E1. ② IL-17A upregulates Runx2 and osterix expression to promote mice calvarial osteoblast to differentiate into mature osteoblast, whereas downregulates Runx2 and osterix expression to inhibit rat calvarial osteoblast to differentiate into mature osteoblast. ③ IL-17A promotes the secrete of IL‐6 and IL‐1β to activate the AKT, STAT3, and ERK1/2 pathways and promotes osteoblastic differentiation of bone marrow mesenchymal stem cells (BM-MSCs), whereas IL-17A inhibits osteoblastic differentiation through inhibiting the Wnt signaling pathway.④ IL-17A promotes human BM-MSCs (hBM-MSCs) to differentiate into osteoblasts by upregulating Runx2 expression. IL-17A with bone morphogenetic protein-2 (BMP-2) or with tumor necrosis factor (TNF)-α synergistically enhance osteogenic differentiation. However, IL-17A inhibits osteoblastic differentiation of hBM-MSCs by inhibiting the Wnt signaling pathway. ⑤ IL-17A promotes bone-derived cells to differentiate into osteoblasts by activating the JAK2-STAT3 signaling pathways.

Slightly confusingly, the effects of IL-17A on osteoblastic differentiation *in vitro* are fraught with contradictions. A study showed that IL−17A promoted the differentiation of murine pre-osteoblastic MC3T3−E1 through the phosphoinositide 3-kinase-serine/threonine kinases (PI3K-AKT) pathway, whereas another study showed that IL−17A of the same concentration inhibited osteoblastic differentiation of MC3T3‐E1 (54, 55). IL-17A can cause an increase in the osteoblastic differentiation of murine calvarial osteoblasts by up-regulating the expression of genes involved in osteoblastic differentiation including Runx2, ALP, osterix, osteocalcin and type I collagen (Colla1), osteoprotegerin (OPG), bone sialoprotein (Ibsp), and osteopontin (Spp1) (43, 44). However, IL-17A inhibits osteogenic differentiation of rat calvarial osteoblast cells by down-regulating expression of genes involved in osteoblastic differentiation including Runx2, ALP, osterix, osteocalcin and type I collagen (56, 57). Different species lead to the expressed differential of IL-17R, which might partly explain this opposite effect (56). When activated by IL-17A, mice bone marrow mesenchymal stem cells (BM-MSCs) secrete IL‐6 and IL‐1β, thereby activating the AKT, STAT3, and ERK1/2 pathways to promote osteoblastic differentiation (55). However, IL-17A inhibits the Wnt signaling, resulting in reduced levels of osteoblast differentiation markers (osterix and osteocalcin) and early osteocyte markers (Dmp1 and Phex), thereby inhibiting osteoblastic differentiation of BM-MSCs (52). Moreover, IL-17A increases the expression of N-cadherin to inhibit PTHR1-LRP-6 interaction in osteoblasts, which also can inhibit the Wnt-signaling pathway (58) (**Figure 2**).

Studies involving human pre-osteoblasts have indicated that IL-17A could promote bone-derived cells to differentiate into osteoblasts through JAK2/STAT3 signaling (59). In addition, IL-17A promotes the differentiation of hBM-MSCs into osteoblasts and promotes the mineralization of osteoblasts by upregulating bone formation-related gene ALP and Runx2 (39). The synergistic effects of IL-17A and bone morphogenetic protein-2 (BMP-2) promote the osteogenic differentiation of hBM-MSCs (60). Besides, the synergistic effects of IL-17A and TNF-α enhance osteogenic differentiation and mineralization of hBM-MSCs by down-regulating Dickkopf-1 (DKK-1), an inhibitor of the Wnt-signaling pathway (61). Osteoblasts and adipocytes are differentiated from a common pluripotent precursor, the mesenchymal stem cell (MSC). Many studies have suggested the differentiation decision of osteoblasts and adipocytes is delicately balanced and may even have a competitive relationship. IL-17A may steer mesenchymal stem cells into an osteogenic fate. IL-17A activates COX-2-induced prostaglandin E2 to inhibit lipid-related proteins include PPAR-γ, FABP4, and adiponectin. Therefore, the differentiation of hBM-MSCs into adipocytes is reduced (62). However, one study indicated that IL-17A inhibited osteogenic differentiation with up-regulated expression of the Wnt antagonist secreted frizzled-related protein 1 (sFRP1) and down-regulated expression of Wnt3 and Wnt6 in hBM-MSCs (63) (**Figure 2**).

The *in vitro* effects of IL-17A on osteoblasts are difficult to be defined. The effects of IL-17A on osteoblasts may not depend on the concentrations. IL-17A probably exerts distinct roles depending on the *in vitro* model used to assess osteoblast development. In addition, different species may also be partly responsible for the controversial results. It is not excluded that the different experimental methods also influence results. To achieve the precise effects of IL-17A on osteoblasts, the type of *in vitro* model, the correspondence between *in vitro* or *in vivo* effects, and the similarity of the effects between animal models and humans should be considered.

## Effects of IL-17A on Bone Disease

The knockout of IL-17A or its receptors in animal models does not affect bone mass, osteoclast numbers, or osteoblast numbers (34, 40, 64–66). Moreover, neutralizing antibodies directed against IL-17A in wild-type mice also do not influence bone mass (65). These results indicate that IL-17A might not have any effect on bone under normal physiological conditions, and it only plays a role in inflammatory conditions or injury. The involvement of IL-17A in immune-mediated bone disease is worthy of exploration.

### Postmenopausal Osteoporosis

Women undergoing natural menopause often experience postmenopausal osteoporosis (PMOP) with a decrease in bone mineral density (BMD) and an increased risk of fractures (67). Estrogen deficiency is the pivotal reason for PMOP. Estrogen deficiency increases osteoclast formation by increasing the number of hematopoietic progenitors and recruiting osteoclast progenitors. Likewise, estrogen deficiency allows prolonged survival of osteoclasts, and the net increase in bone resorption leads to bone loss (68, 69). Recent studies show that osteoimmunology is involved in the pathogenesis of PMOP. Furthermore, T-cell activity is increased while B-cell activity is decreased in postmenopausal women (70, 71). Estrogen deficiency can activate T cells and promotes the production of a variety of immune factors. These factors include IL-6 (72), TNF-α (73), IFN-γ (74), IL-1β, and TNF-β (75), all of which enhance bone loss.

Despite one study showing that the level of serum IL-17A in postmenopausal women with low BMD is not significantly different from that in women with normal BMD (76), other studies have indicated that postmenopausal women with osteoporosis have a higher concentration of serum IL-17A, and have more peripheral blood IL-17-producing CD4^+^ T-cells (58, 77–80). In postmenopausal women with osteoporosis, the concentration of serum IL-17A is negatively correlated with BMD, but is positively correlated with sRANKL level (78, 79).

In animal studies, ovariectomy (OVX) causes estrogen deficiency and bone loss. The drastic reduction of estrogen increases expression of the differentiation factors of Th17 including STAT3, ROR-α, and ROR-γt, which indicates that more peripheral blood mononuclear cells can differentiate into Th17 and produce IL-17A (80). The level of IL-17A in the bone marrow and blood are increased after OVX (38). IL-17RA knockdown and anti-IL17 antibody injection both protect against bone loss caused by estrogen deficiency (40). Anti-IL-17 antibodies and parathyroid hormone (PTH) can be used in combination to further protect OVX-induced bone loss (58, 81). Anti-IL17 antibodies exert a bone protective effect by inhibiting osteoclast formation, decreasing the apoptosis of osteoblasts and promoting the formation of mineralized nodules. Moreover, the blocking of IL-17A may inhibit osteoblasts to produce osteoclastogenic factors including TNF-α, IL-6, and RANKL in OVX mice (38, 40). Interestingly, anti-IL-17A antibodies have also been reported to reverse the higher frequency of CD4^+^ T cells and the proliferation of B220^+^ cells in bone marrow caused by estrogen deficiency. Anti-IL-17A antibodies exert an immuno-protective effect and translate to superior skeletal outcomes (81).

### Rheumatoid Arthritis

RA is an autoimmune disease characterized by the upregulation of various immune factors that recruit and activate various immune cells, especially T and B cells to destroy cartilage and bone (82). RA patients have higher levels of IL-17A in synovial tissue and fluid compared with normal subjects (83–85). In a 2-year prospective study, the expression of IL-17A in synovial tissues was associated with increased joint damage progression in RA (86). Except for synovial tissue, RA patients have a higher concentration of serum IL-17A, which is proportional to the severity of RA (87–90). Moreover, the PBMCs of patients with RA produce more IL-17A (91). The increased levels of IL-17A in synovial fluid, serum, PBMCs are associated with the Disease Activity Score of 28 joints (DAS28), and levels of C-reactive protein (CRP), the erythrocyte sedimentation rate (ESR), and rheumatoid factor (RF) expression (92, 93). In addition, evidence suggests that IL-17A is not only related to the progression of the disease but is also associates with the occurrence of the disease. Studies indicate that IL-17A plays an important role in the pre-onset, early, and chronic stages of RA (94, 95).

Collagen-induced arthritis (CIA) is the most common animal model for studies involving RA (96). High levels of IL-17A are detected in CD4^+^ T cells and γδT cells located in joints of CIA mice (97). Th17 cells are localized adjacent to osteoclasts in the subarticular cartilage and express IL-17A, indicating the involvement of IL-17A in bone destruction of CIA (97). Local injection of IL-17A in the joint increases the morbidity of CIA and joint damage, while local injection of an adenoviral vector expressing murine IL-17A in the joint also accelerates the initiation of CIA and inflammation (98). Treatment with a soluble IL-17R fusion protein or anti-IL-17A antibody prevents bone erosion and the initiation of CIA (99, 100). In the progression of CIA, the local injection of IL-17A in knee-joint promotes arthritis and exacerbates joint damage (101). Anti-IL-17A antibodies ameliorate the severity of arthritis, cartilage damage, and bone loss (97, 102). Combinations that neutralize both TNF-α and IL-17A can also alleviate CIA progression (103). The combination of anti-IL-1β and anti-IL-17A antibodies significantly reduce the severity of arthritis, alleviates bone and cartilage damage, and down-regulates IL-1β, IL-6, IL-17A, IFN-γ, RANKL, and MMP-3 (104, 105). IL-17A plays an important role not only in the pathogenesis but also in the progression of the disease. Moreover, IL-17A is involved in the pathological process of bone erosion and bone loss.

The pathological mechanism of IL-17A may involve the immune activation and an immune cascade reaction in RA. In addition, the activation of osteoclasts promotes bone erosion in RA. Collagen-specific T cells and collagen-specific IgG2a are involved in the development of CIA. IL-17A is responsible for the priming of collagen-specific T cells and collagen-specific IgG2a production (106). Anti-IL-17A significantly reduces splenocytes proliferation and reduces leukocyte recruitment in CIA (105, 107). Anti-IL-17A also down-regulates IL-1β, IL-1, IL-6, IL-17A, and IFN-γ in the joint (104, 105). Increased osteoclast activity in the subchondral, trabecular, and cortical bone erosion areas is observed after local IL-17A overexpression in joint (98, 101, 102).

Several drugs targeting IL-17A are currently being evaluated in clinical trials, but the benefit seems to be not satisfactory for RA. Brodalumab, a human anti-IL-17 receptor A (IL-17RA) monoclonal antibody, did not demonstrate clinical efficacy in active RA patients (108). The humanized anti-IL-17A monoclonal antibody ixekizumab improved the signs and symptoms of RA patients in a phase II study, but the efficacy was not considered robust sufficient to support continued development (109). Bimekizumab is a monoclonal antibody that selectively neutralizes IL-17A and IL-17F. Bimekizumab plus certolizumab pegol further reduced disease activity score 28-joint count C-reactive protein (DAS28(CRP)) for RA patient in a phase II study, but more messages about the efficacy and safety is lack (110). Secukinumab, a fully human monoclonal antibody directed against IL-17A, has advanced in phase III studies. Secukinumab achieved 20% improvement in the American College of Rheumatology criteria (ACR20) at week 24 among patients with active RA, although, studies have suggested that secukinumab may not provide additional benefit beyond the currently approved therapies to such patients and further development was not pursued due to lack well-pleasing efficacy (111–114).

### Psoriatic Arthritis

PsA is an immune-mediated chronic inflammatory arthritis associated with psoriasis. PsA presents synovial inflammation, bone destruction, and juxta-articular new bone formation (115, 116). Aberrant cytokine expression of TNF-α, IL-23, IL-22, IL-9, IL-15 is involved in the pathological mechanisms of PsA (117). Serum IL-17A levels are higher in psoriasis patients (118). IL-17^+^ CD4^+^ T cells and IL-17A secretion increase in peripheral blood and synovial fluid of PsA (119, 120). Besides CD4^+^ T cells, IL-17A-producing ILCs are present in the synovial fluid of PsA (121). IL-17A^+^CD8^+^ T cells are enriched in the joints of patients with PsA and have been correlated with disease activity and bone erosion (7).

In the animal model of PsA, increased serum IL-17A is associated with bone loss. The imbalance between osteoblasts and osteoclasts is the main cause for the appearance of PsA in the bone. Skin-resident cells such as keratinocytes, γδT cells, and innate lymphoid cells express IL-17A, which inhibits osteoblasts and osteocytes function through the Wnt signaling (52). In addition, IL-17A may also promote epidermal sheet, keratinocytes and skin resident T cells to produce RANKL (122).

Clinical trials of antagonizing IL-17A in PsA are underway. Secukinumab improves the signs and symptoms of active PsA (123). At the same time, secukinumab inhibits the progression of bone erosions and maintains bone stability (124–127). In 2016, secukinumab became the first targeting IL-17A drug approved by the FDA for the treatment of active PsA. Ixekizumab, an IL-17A specific monoclonal antibody, improved the signs and symptoms of patients with active PsA and inhibited bone damage progression in PsA (128, 129). In 2017, ixekizumab was approved by the FDA for the treatment of PsA. Brodalumab, a fully human monoclonal antibody targeting the IL-17 RA, achieved ACR20 at week 16 among patients with PsA in a phase III study (130). However, the trials were terminated early due to a possible safety concern about suicidal ideation and behavior (131). Bimekizumab, which inhibits both IL-17A and IL-17F, improved ACR50 in patients with active PsA in a phase II trial and phase III trials that are currently underway (132).

### Axial Spondyloarthritis

Axial spondyloarthritis (axSpA) is chronic inflammatory bone diseases including non-radiographic axial spondyloarthritis (nr-axSpA) and radiographic axial spondyloarthritis (ankylosing spondylitis [AS]). Bone destruction and new bone formation may occur simultaneously in axSpA. Various types of cytokines including IL-17A, TNF-α and IL-23 are involved in the pathological processes (133, 134). Many studies have indicated that IL-17 is involved in immunopathogenesis of axSpA (135). IL-17^+^ CD4^+^ T cells increase in peripheral blood of axSpA and IL-17A synthesis also increases (120, 136–138). Levels of IL-17A in the synovial fluid are elevated in patients with AS (59). Serum IL-17A levels are also higher in AS and elevated IL-17 serum levels may associate with the development of AS (139, 140). A few studies have focused on the role of IL-17 in the processes of axSpA bone damage. IL-17A promotes local mesenchymal stem cell populations to osteoblast differentiation and increases mineralization in AS by JAK2/STAT3 signaling, which may be a mechanism of ankyloses progression (59). Anti-IL-17A treatment prevented bone loss and induced new bone formation in an animal model of pathogenic SpA, mycobacterium tuberculosis-induced disease in B27/hβ2m-transgenic rats (141).

Several IL-17A targeted drugs are currently in clinical trials. Secukinumab and Ixekizumab are both anti-interleukin-17A monoclonal antibodies and have been reported to improve the signs and symptoms of axSpA (142–148). To date, the FDA has approved both antibodies for the treatment of adults with active AS and nr-axSpA with objective signs of inflammation. Netakimab, a humanized monoclonal antibody targeting IL-17A, significantly achieved 20% improvement in Assessment of Spondyloarthritis International Society (ASAS20) response among patients with AS in a phase II study (149). Bimekizumab, a monoclonal antibody that selectively neutralizes IL-17A and IL-17F, achieved ASAS40 response at week 12 in a phase II trial (150). Phase III trials that aim to assess the efficacy and safety of netakimab and bimekizumab in AS patients are currently underway.

## Conclusion and Perspectives

IL-17A is involved in innate immune responses and adaptive immunity. Meanwhile, IL-17A plays an important role in bone homeostasis *via* activation of complex cellular and molecular interactions. IL-17A may exert direct positive or negative effects on osteoclastogenesis depending on its concentration *in vitro*. Osteoblasts are most closely associated with osteoclasts, which both are involved in bone metabolism. IL-17A indirectly regulates osteoclastogenesis by inducing multiple factors derived from the osteoclast-supporting cells. The effects of IL-17A on osteoblasts may depend on the different experimental models of osteoblast development and species tested *in vitro*. These aforementioned cell studies provide evidence supporting the skeletal-regulatory properties of IL-17A and support the concept that IL-17A acts as the link between the skeletal and the immune systems. Future research should focus on the molecular pathways involved and explore the precise reasons for the dual effects of IL-17A in bone cells.

Mechanistic studies have hinted that IL-17A is a bone-destroying cytokine involved in immune-mediated bone diseases, such as PMOP, RA, PsA, and axSpA. IL-17A exerts a negative effect on bone by promoting osteoclastogenesis, excessively activates bone formation, and initiates an immunologic cascade. Indeed anti-IL-17A therapy has produced promising results in clinical trials of RA, PsA, and axSpA, although, few studies have focused on bone damage. A deeper understanding of the molecular mechanisms of IL-17A involved in bone disease may supply novel therapeutic interventions and provide a new thought to prevent bone loss and osteoporosis associated with immune-mediated bone diseases.

## Author Contributions

XY provided the conception of the manuscript. MT and LL were contributed to perform the literature search and drafted the work. All authors contributed to the article and approved the submitted version.

## Funding

This work was supported by grants from the National Natural Science Foundation of China [No. 81770875]; the Sichuan University [No. 2018SCUH0093]; the Post-Doctor Research Project, West China Hospital, Sichuan University [No.19HXBH053]; the Health and Family Planning Commission of Sichuan Province [No. 19PJ096]; and the 1.3.5 project for discipline of excellence, West China Hospital, Sichuan University [No. 2020HXFH008, No. ZYJC18003]; the National Clinical Research Center for Geriatrics of West China Hospital (No. Z2018B05).

## Conflict of Interest

The authors declare that the research was conducted in the absence of any commercial or financial relationships that could be construed as a potential conflict of interest.
